# Transcriptome of Sterile Testes in *dnd*-Depleted Atlantic Salmon (*Salmo salar* L.) Highlights Genes Involved in Gonadal and Brain Development

**DOI:** 10.3390/genes16091095

**Published:** 2025-09-16

**Authors:** Aleksei Krasnov, Sergey Afanasyev, Jens-Erik Dessen, Marianne H. S. Hansen, Marianne Vaadal, Helge Tveiten, Øivind Andersen

**Affiliations:** 1 The Norwegian Institute of Aquaculture, Nofima, 9291 Tromsø, Norway; jens-erik.dessen@nofima.no (J.-E.D.); marianne.vaadal@nofima.no (M.V.); oivind.andersen@nofima.no (Ø.A.); 2 Sechenov Institute of Evolutionary Physiology and Biochemistry, St. Petersburg 194233, Russia; afanser@mail.ru; 3 Norwegian College of Fishery Science, The Arctic University of Norway, 9019 Tromsø, Norway; helge.tveiten@uit.no

**Keywords:** Atlantic salmon, testes, transcriptome, dead end, reprogramming

## Abstract

Background/Objectives: Inactivation of the *dnd* gene involved in the development of primordial germ cells (PGCs) leads to the loss of gametes and halts reproductive development. Studies on sterile fish allow for the identification of genes and processes associated with GC differentiation. Methods: Atlantic salmon with GC-ablated testes were produced by temporal silencing of *dnd*. Gene expression was analyzed in sterile and fertile testes using 44k microarray and qPCR. Results: In sterile testes, transcripts of several GC markers were detected at low levels, suggesting the presence of cells with a GC-related expression profile that failed to initiate spermatogenesis. Expression of 260 genes was undetectable in the gonads of sterile males and females, and 61.5% of these were also inactivated during first maturation of fertile testes. This group was enriched with genes highly expressed in the brain, including those involved in endocrine and paracrine regulation, synaptic transmission, and numerous genes critical for brain development; among them, 45 genes encoding homeobox proteins. Another group of 229 genes showed increased expression in developing testes and included genes involved in neurosecretion and brain development regulation. GC-ablated testes showed increased expression of reproductive regulators such as *amh* and *sdy* and numerous immune genes, suggesting a reprogramming of GC-depleted testes. Temporal silencing of *dnd* indicated common developmental processes in the brains and gonads of Atlantic salmon testis that become inactive in testes at first maturation. These processes may play roles in PGC homing, the creation of a specific environment required for spermatogenesis, or facilitating communication between the gonads.

## 1. Introduction

Development of gametes is initiated by speciation of primordial germ cells (PGC) in early embryonic development followed by their migration to the genital ridge to form gonad anlagen together with somatic cells. The somatic cell lineages then develop into Sertoli cells and Leydig cells in the testis, and germline stem cells sustain gamete production giving rise to the sperm. PGC and descending cells express a suite of genes required for maintenance of pluripotency, prevention of premature differentiation, and preservation of genome integrity. The RNA-binding protein dead end (*dnd*) protects PGCs from reprogramming into other cell types by inhibiting the interaction of microRNAs with the target messenger RNAs [1]. Accordingly, dnd-depleted PGCs in zebrafish lost their characteristic morphology and adopted specific somatic cell fates, whereas only a few were found to undergo apoptosis [2]. PGC-specific expression of *dnd* has been shown in various fish species [3,4,5,6]. Germ cells are a prerequisite for female development in zebrafish (*Danio rerio*), medaka (*Oryzias latipes*), and mackerel (*Scomber australasicus*) as ablation of PGCs results in phenotypic, though sterile, males only [7,8,9,10,11]. In comparison, PGC ablation failed to affect the sexual fate of gonadal somatic cells in loach (*Misgurnus anguillicaudatus*), goldfish (*Carassius auratus*), grass puffer (*Takifugu alboplumbeus*), and salmonids, which could develop as either phenotypic males or females [12,13,14,15,16,17].

PGCs have been a focus of developmental biology since their discovery in the XIX^th^ century as reviewed in [18,19]. Commercial aquaculture requires effective methods of sterilization to prevent early maturation, improve flesh quality, and reduce gene flow from farm escapees to wild fish [20]. Promising results have been achieved by transient degradation of *dnd* transcripts or by permanent inactivation of the *dnd* gene using CRSPR Cas [12,21,22]. Sterilization of Atlantic salmon by temporal knockdown of *dnd* did not block development of the somatic testes, and the GC ablated fish maintained endocrine regulation by the hypothalamus–pituitary–gonad (HPG) axis during maturation entrance, but without any further progression of the reproductive cycle [23]. In addition to achieving a practical goal, GC-ablated gonads may serve as a powerful research tool to highlight genes specifically expressed in GC and structures that critically depend on interactions with the germline. In this study, we present a transcriptomic analysis of GC-ablated Atlantic salmon males. Genes that were undetectable or differentially expressed in the testes of sterile males were categorized based on their involvement in normal developmental processes, such as gonadal growth and the initiation of sexual maturation. The dataset was integrated with transcriptomic data from testes of males entering the first reproduction cycle, obtained from a study that utilized the same genome-wide Atlantic salmon microarray platform [24].

## 2. Materials and Methods

### 2.1. Fish Material

Gene expression analyses were performed on testes from Atlantic salmon described in detail in [23]. In brief, *dnd* knockdowns were produced by microinjection of “Gapmer” antisense oligonucleotides targeting the *dnd* mRNA at the one-cell stage [25]. Embryos, alevins and juveniles were raised under standard hatchery conditions [23]. Sterility caused by germ cell ablation was assessed by regular histology, in situ hybridization, qPCR, and immunohistochemistry of the germ cell marker vasa (ddx4) during the embryonic (c. 500-degree days) and several juvenile stages [23]. Fish used in the present work were kept at a photoperiod of daily 6 h light (6L:18D) and water temperature of 5–6 °C for eight weeks followed by continuous light and 10 °C for six weeks according to established protocols of smoltification control. Samples of *dnd* knockdowns (referred to as sterile in text) and fertile controls were collected at the onset of constant light (fish weight of 220–250 g) and six weeks later (fish weight of 350–400 g) are referred to as w0 and w6, respectively. The study did not involve any treatments that could cause suffering. For sampling purposes, fish were euthanized using an overdose of tricaine (Merck, Rahway, NJ, USA).

### 2.2. RNA Extraction

Tissue samples were stored in RNALater (Thermo Fisher Scientific, Waltham, MA, USA). Total RNA was extracted using an automated Biomek 4000 (Beckman Coulter, Brea, CA, USA) according to the manufacturer’s protocol for this application. A NanoDrop ND-8000000 Spectrophotometer (NanoDrop Technologies, Wilmington, DE, USA) and 2100 Bioanalyzer with RNA Nano Chips (Agilent Technologies, Santa Clara, CA, USA) were used for assessment of, respectively, concentration and integrity of RNA (RIN values > 7.5).

### 2.3. Microarray

Transcriptome analyses used genome-wide 44 K Atlantic salmon DNA oligonucleotide microarray Salgeno-2 (GPL28080) containing 60-mer probes to all identified protein coding genes. RNA (220 ng per reaction) was amplified, labeled, and fragmented using a One-Color Quick Amp Labelling Kit and a Gene Expression Hybridization kit (Agilent Technologies, Santa Clara, CA, USA), and 1650 ng of cRNA was loaded on the array. After overnight hybridization in an oven (17 h, 65 °C, rotation speed 0.01× *g*), the arrays were washed with Gene Expression Wash Buffers 1 and 2 and scanned with SureScan (Agilent Technologies, Santa Clara, CA, USA). Microarray analyses were performed on 5 sterile and 6 fertile testes at w0, and on 12 sterile testes and 12 fertile controls at w6. Results were analyzed with bioinformatic pipeline STARS [26]. To investigate the developmental profiles of genes affected by *dnd* silencing, this dataset was integrated with microarray analysis results from Atlantic salmon males undergoing sexual maturation [24]. Differentially expressed genes (DEG) were selected by the following criteria: log2-ER (expression ratio) > 0.8 (1.75-fold) and *p* < 0.05. For enrichment analysis, the number of genes associated with each term was compared between the list of DEGs and the full microarray platform, with statistical significance assessed using Yates’ corrected Chi-square test. Functional categories and pathways were ranked based on enrichment (fold), *p*-values, and the number of DEGs. Functional groups of genes (STARS annotation) with coordinated expression differences were identified by significant deviation of mean log_2_-ER from zero baseline. Additionally, a body map of normalized transcriptomes generated using the Salgeno microarray [27] was utilized to assess gene expression distribution in 21 different cell types and tissues of Atlantic salmon. Expression levels were quantified as dSI (normalized relative signal intensity). Results of microarray analyses were submitted to NCBI GEO Omnibus (GSE297806). In addition to testes, the submission includes data from ovary, brain, pituitary, gills, and head kidney of the same fish.

### 2.4. Quantitative RT qPCR

Expression of 12 DEGs in sterile and fertile testes was verified with PCR at w6. Primers (Table 1) were designed using Primer 3. The PCR products were purified and sequenced by Eurofins to confirm amplification of correct targets. The transcript abundance was analyzed using a QuantStudio5 instrument (ThermoFisher, Waltham, MA, USA), following the MIQE guidelines [28]. Each reaction was run in duplicate and included non-template and non-RT enzyme controls for each gene. The PCR master mix was prepared with 0.5 μL forward, 0.5 μL reverse primer (Table 1), final concentrations of 0.5 μM and 5 μL SYBR Green (Applied Biosystems, Waltham, MA, USA). This master mix was added to a 4 μL of a 1:10 dilution of cDNA per well, resulting in a total reaction volume of 10 μL. The PCR protocol included an initial incubation at 95 °C for 20 s, followed by 40 cycles of denaturation at 95 °C for 1 s and annealing at 60 °C for 20 s, and finally, one last step of extension with an increase of 0.075 °C per second until 97 °C. Primer efficiency was assessed using 10-fold serial dilutions of cDNA for each primer set, and the specificity of amplification was verified by melting curve analysis. The geometric means of *rpol2* and *eif-3* were used for normalization. These reference genes were selected due to their stable expression in Atlantic salmon [29].

## 3. Results

### 3.1. Gene Markers of Germ Cells

**Figure 1:**
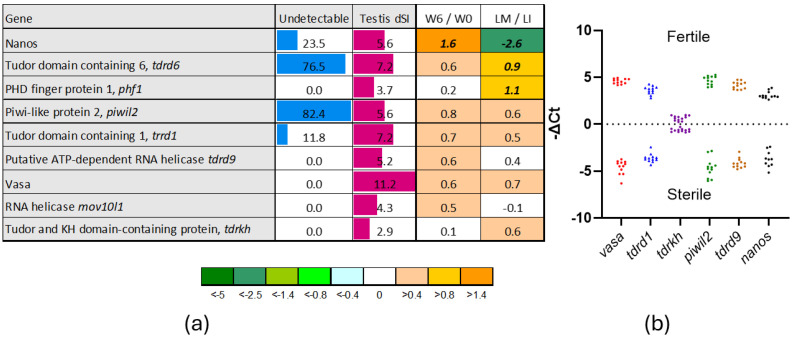
Gene markers of germ cells. (**a**) Microarray. Panel presents percentage of sterile testes in which expression was not detected; expression in relation to Atlantic salmon tissues (dSI, body map) and changes (log2-ER) during gonadal growth (w6/w0) and maturation of large fish (LM/LI). Differential expression is indicated with bold italics. (**b**) RT-qPCR of selected GC markers in sterile and fertile testes (centered ΔCt). *Dnd* was not detected in sterile testes.

Expression of nine GC gene markers was detected in sterile testes (Figure 1a). Five of these genes were expressed in all analyzed samples, whereas *tdrd6* and *piwil2* were undetectable in 76.5% and 82.5% of cases, respectively. *Tdrd6* is involved in spermatogenesis through RNA processing [30], while *piwil2* plays a role in germline stem cell maintenance and transposon silencing [31]. Similar functions are attributed to *tdrd1*, *tdrd9*, *tdrkh*, and *mov10l1*, while *phf1* is involved in chromatin modification and epigenetic regulation [32]. Six of these GC gene markers were analyzed with RT-qPCR (Figure 1b), which did not detect *dnd* transcripts in sterile testes, whereas the remaining genes showed significantly higher expression in fertile testis, with differences ranging from 2.4- to 570-fold. *Nanos*, which prevents untimely maturation of GC, was upregulated in growing testes but showed decreased expression at maturation (Figure 1a). In contrast, *tdrd6* and *phf1* were stimulated during this period. Other GC gene markers, such as *piwil2*, *tdrd1*, *tdrd9*, *vasa*, *mov10l1*, and *tdrkh* did not exhibit developmental changes above the threshold for differential expression. *Vasa*, a key regulator of RNA metabolism in GC, displayed the greatest tissue specificity, with a dSI of 11.2—one of the highest among all Atlantic salmon genes. Other GC markers shown in Figure 1a are characterized by preferential expression in the gonads, although they were also active in other tissues.

### 3.2. Genes Undetctable or Downregulated in Sterile Testes

#### 3.2.1. Undetectable Genes

**Figure 2:**
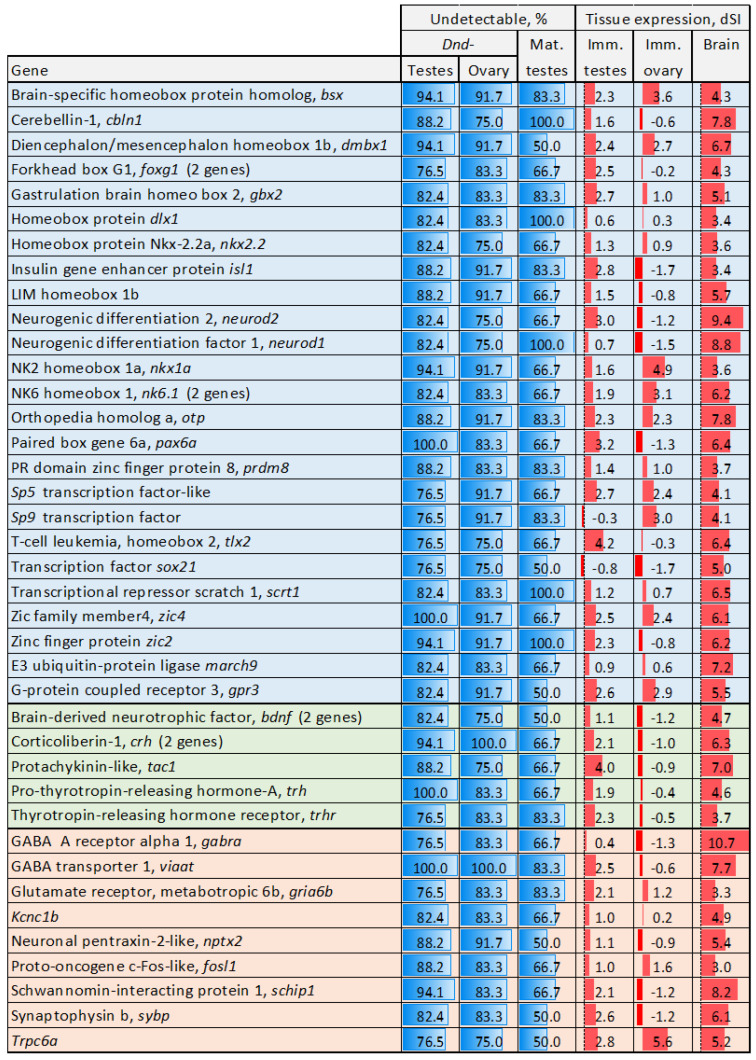
Genes undetectable in at least ¾ of the sterile GC-depleted testes and ovaries and ½ of fertile maturing testes. The percentage of samples lacking transcripts is shown. dSI reflects the expression levels in normal testes and brain in relation to Atlantic salmon tissues (body map). Color highlights genes associated with developmental (light blue) and endocrine (green) processes, and genes involved in neural system function (light red).

Microarray analyses in Atlantic salmon typically reveal decreased transcript levels rather than a complete absence of signal. A distinctive feature of this experiment was the undetectable expression of a substantial number of genes in GC-ablated gonads, in both males and females. Transcripts of 260 genes were not found in at least 75% of sterile testes and ovaries, and 165 of these genes were also inactivated during the maturation of fertile testes (Table S1). Nearly all GO terms enriched among this gene set are associated with brain and nervous system development (Table S2), and 38% of genes exhibited preferential expression in the Atlantic salmon brain (dSI > 3). Of note was the large number of genes encoding proteins with homeobox domains (45 genes). Genes with essential roles in brain development and function are shown in Figure 2. Several genes are specifically involved in early brain development and regionalization, including *atp*, *bsx*, *dlx1*, *nkx1a*, *nk6.1*, *gbx*, *gsc*, and *otp* [33,34,35,36]. Others control neural patterning at various levels: *zic2* and *zic4* are required for neural tube closure [37], *dmbx1* is crucial for spinal cord formation [38], and *tlx2* regulates neural crest development [39]. Genes essential for forebrain, midbrain, hindbrain, and cerebellum development include *march1*, *foxg1*, *gbx2*, *dmbx1*, and *cbln1* [40,41,42]. Additionally, *pax6a* is a key regulator of eye development [43]. Several genes contribute to neurogenesis and neural differentiation include the following: *sox21* maintains neural stem progenitor cells [44], while *gpr3* and *neurod1* and *2* regulate neurogenesis. *Scrt* is involved in neuronal differentiation. *Isl1* is critical for motor neuron development [45], *nkx2-2* for oligodendrocyte differentiation [46], and *sp5/9* for interneuron formation. *Bdnf*, *nptx2*, and *sybp* participate in excitatory neurotransmission and synaptic plasticity [47], whereas *viiat* is responsible for packaging GABA (γ-aminobutyric acid) and glycine into synaptic vesicles in inhibitory neurons [48]. *Schip1* regulates myelinization, while *bsx* is implicated in hypothalamic development and behavioral control [49]. *Fosl1* is a marker of neuronal activity [50]. Several genes have endocrine functions. *Crh* and *trhr* play a central role in the hypothalamic–pituitary–adrenal (HPA) axis [51]. *Tac1* encodes a precursor to several neuropeptides and *trh* is involved in neuroendocrine signaling. RT-PCR analysis was performed on six selected genes (Table 1) to validate microarray results. As expected, transcripts of *viaat*, *hr6*, *soho*, *gbh*, *dbx1*, and *crf1* were detected in all fertile but in none of the sterile testes.

#### 3.2.2. Genes Downregulated in Sterile GC-Depleted Testes

**Figure 3:**
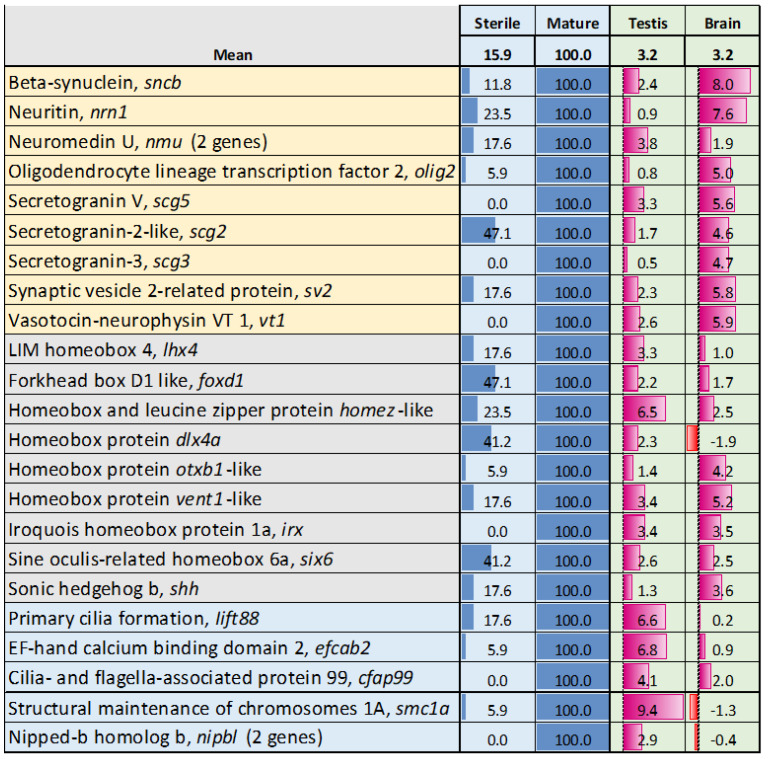
Genes with increased expression during normal gonadal growth from w0 to w6 that were downregulated in sterile testes. The percentage of samples lacking detectable transcripts is shown. dSI reflects the expression levels in normal testes and brain in relation to Atlantic salmon tissues (body map). Color highlights genes associated with nervous system (yellow), development (gray), and reproduction (light blue).

This group comprises 229 genes that were downregulated, but not below the detection threshold, in sterile testes and showed increased expression during gonadal growth. It was also enriched for genes associated with the nervous system, including homeobox genes (Figure 3). Among these, *six6*, *lhx4*, *homez*, and *irx* play crucial roles in the development of the hypothalamus, pituitary gland, hippocampus, and thalamus [52,53,54]. *Lhx4* regulates retinal neurons differentiation and vision [55]. *Dlx4a* is important for differentiation of GABAergic sensory neurons [56]. Genes involved in neuronal and neuroendocrine signaling (highlighted in the upper part of Figure 3) exhibit notably high relative expression in the brain. Genes with key roles in the neuroendocrine regulation of reproduction include vt1, *olig2*, *nmu*, *nrn1*, *sv2*, *scg2*, *scg3*, and *scg5* [57,58,59,60,61]. A hallmark of this group was the high proportion of zinc finger (zf) proteins, accounting for 21.4% of the total. Unlike many homeobox genes, zf did not show preferential expression in testes and brain of Atlantic salmon (mean dSI, respectively, 2.6 and −0.3). Several genes linked to specific reproductive and developmental processes including *smc1a* and *nipbl*—three genes (meiosis), *lift88* and *cfap99* (cilia formation) and *efcab2* (sperm motility) were downregulated but not inactivated in sterile GC-ablated testes.

### 3.3. Genes with Stable Expression During Gonadal Growth and Maturation

Stable expression without significant changes in normal testes during development was observed in 1067 genes that were downregulated in sterile gonads. Enrichment analysis primarily identified functional groups involved in core cellular processes, including chromosome maintenance, DNA replication, cell division, transcription, and RNA degradation, as well as nucleotide and energy metabolism (Table 2). Additionally, processes such as double-strand break repair, base excision repair, and gene silencing by RNA are likely associated with spermatogenesis.

### 3.4. Genes with Expression Changes at Maturation

**Figure 4:**
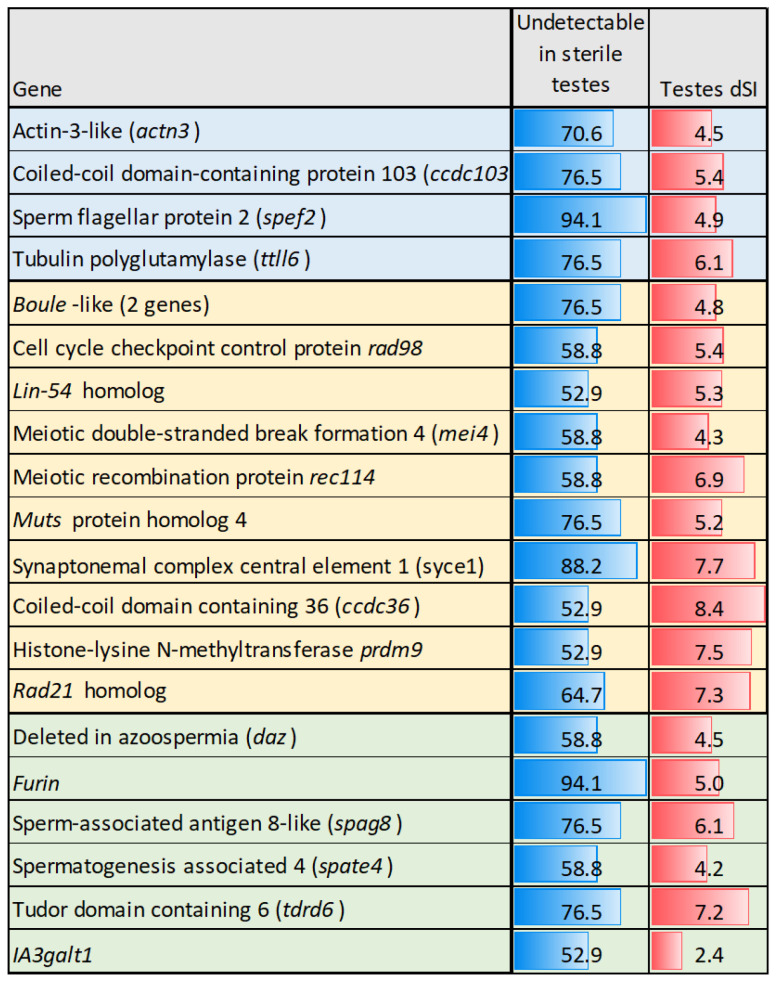
Genes undetectable in not less than ½ of sterile testes that were upregulated during sexual maturation. Color highlights genes associated with cilia structure and activity (light blue), DNA recombination (yellow), and regulation of spermatogenesis (light green). dSI reflects expression levels in normal testes in relation to Atlantic salmon tissues (body map).

Increased and decreased expression was observed in 790 and 689 genes, respectively, when comparing maturing to immature testes. The enriched functional terms with highest ranks are listed in Table 3. This group included genes involved in notochord and brain development that were downregulated but not completely in GC-depleted and maturing testes. The term “DNA replication” was enriched in both up- and downregulated genes, reflecting changes in the repertoire of genes involved in this process during the transition from immature to mature testes. Maturation was primarily associated with cell division, particularly meiosis, as well as DNA repair, chromosome maintenance, and segregation. Enrichment of homologous recombination (KEGG pathway) in downregulated genes indicates that this process might be principally completed by the time of sexual maturation. Maturation was marked with a decline in the activity of growth factors and developmental processes. A suite of genes linked to spermatogenesis were inactivated in more than half of sterile GC-depleted testes (Figure 4). *Daz* and *boule* are members of the Deleted in Azoospermia multigene family that regulate mRNA translation in germ cells, influencing the expression of genes critical for meiosis, spermatid differentiation, and sperm maturation [62,63]. *Prdm9* and *rad21* participate in homologous recombination [64,65]. However, transcripts of 35 genes involved in meiosis were detected in sterile GC-depleted testes, and only 2 out of 48 genes associated with cilia were silenced in half or more of sterile males.

### 3.5. Genes Upregulated in Sterile Testes

**Figure 5:**
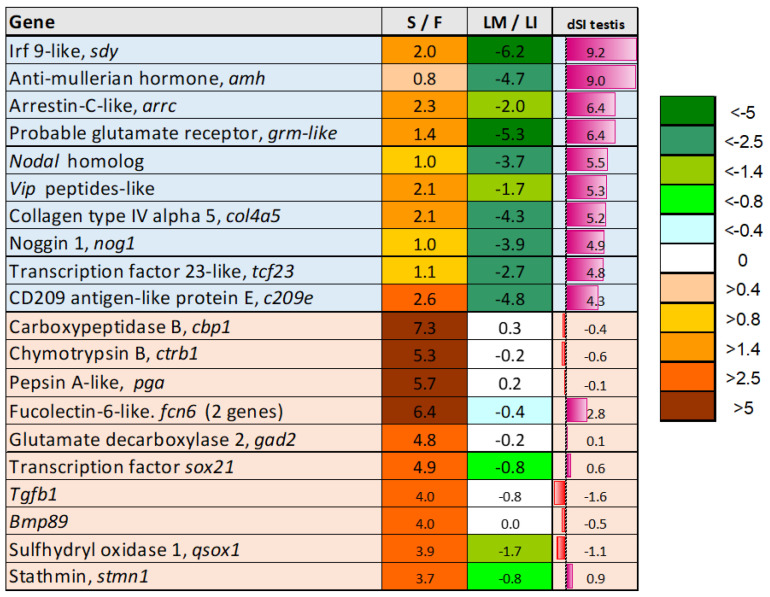
Genes upregulated in sterile testis. Differential expression (log2-ER) of sterile (S) to fertile (F) testes and mature (M) to immature testes (I) of large fish. dSI reflects expression in normal testes in relation to Atlantic salmon tissues (body map). Color highlights testis-specific genes. dSI > 4.3 (light blue) and genes with high expression in sterile testes (light red).

**Figure 6:**
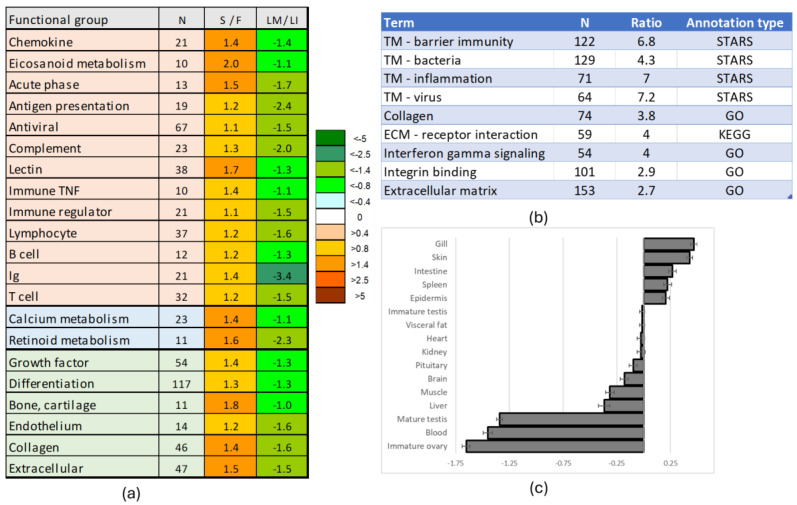
Genes upregulated in sterile testes. (**a**): Functional groups (STARS annotation) with similar expression changes. DEG numbers are indicated, data is mean log2-ER of sterile to fertile gonads (S/F) and maturing to immature testes of large fish (LM/LI). (**b**): Enrichment analysis, most highly ranked terms. (**c**): Expression of genes upregulated in sterile testes in Atlantic salmon tissues and cells (body map, dSI). TM—transcription modules, ECM—extracellular matrix, N—number of genes.

The upper section of Figure 5 highlights testis-specific genes (dSI > 4 or approximately 8-fold higher than the average level) that showed decreased expression at maturation. This small group includes *amh*, a key inhibitor of sexual maturation, and the immune related irf9-like gene named *sdy*, which regulates male sex determination by preventing ovarian development in salmonids [66]. *Vip* may play a role in regulating sex hormone secretion, whereas the reproductive functions of *nodal* and *nog1*, members of the TGF-β and BMP families, respectively, remain unknown. Notably, *tgfb1* and *bmp8B* were among the most upregulated genes, but the greatest expression changes were observed in proteolytic enzymes. Sterility appeared to enhance processes that counteract maturation, but the primary effect of *dnd*-silencing was developmental reprogramming. The major trends included increased expression of immune genes, activation of developmental pathways unrelated to reproduction, and enhanced extracellular matrix deposition (Figure 6a). Specifically, sterile testes showed stimulation of chemokine and eicosanoid signaling, antigen presentation, humoral and cellular immune responses, and an increase in B and T lymphocyte populations. At the same time, enhanced differentiation of bone, cartilage, and endothelium was indicated. These functional groups were downregulated in testes at maturation. Enrichment analysis (Figure 6b) assigned the highest ranks to transcription modules (TMs) associated with bacterial responses and inflammation—gene sets identified through meta-analysis of transcriptome databases [67]. Additionally, genes related to cell adhesion (integrin binding) and extracellular matrix remodeling were markedly enriched (Figure 6b). On average, genes upregulated in sterile testes showed the highest expression levels in the skin and gill, while their expression was lowest in the immature ovary (Figure 6c).

## 4. Discussion

Comparison of sterile and fertile testes in Atlantic salmon revealed numerous genes affected by *dnd* knockdown. Combining these results with profiles from normal gonadal development may provide insights into the fate of germ cells and the regulation of Atlantic salmon reproduction. The upregulated genes indicated the altered fate of the GC-ablated gonad. Sterile testes showed activation of functional groups of genes that are typically downregulated during sexual maturation, primarily those related to immune function. This might suggest gonadal degeneration and resorption, followed by fibrosis, as indicated by increased expression of genes involved in collagen synthesis and other extracellular matrix components. However, no clear transcriptional signature of acute inflammation was observed. Notably, transcripts of genes involved in adaptive immunity, specifically those associated with lymphocytes, B and T cells, and hematopoiesis, were highly expressed. Reprogramming toward an alternative role, with a predominance of immune functions, appears to be the most likely scenario in the development of sterile testes.

As expected, many genes involved in spermatogenesis were downregulated or completely undetectable in sterile testes. However, a suite of GC markers retained low-level expression, along with genes involved in meiosis and the formation and function of flagella. This suggests the presence of a small population of GC-derived cells in sterile testes that have lost the capacity to differentiate into sperm. While temporal *dnd* knockdown did not completely abolish the expression of genes directly related to reproduction, the most pronounced effect was observed in a large set of genes associated with the nervous system, including those expressed in the brain. Transcripts of a significant fraction of these genes were not detected in sterile testes. Interestingly, a striking similarity between the adult human brain and testes was recently revealed through proteomic comparison [68]. Our results suggest that the same genes may be involved in fundamental developmental processes of both organs in Atlantic salmon, with their expression dependent on *dnd* and possibly other germ cell-related genes. Most of the genes involved in brain structure specification that were undetectable in GC-depleted testes are transcription factors, including 45 genes containing homeobox domains. While the roles of homeobox proteins and other developmental regulators may vary across taxa, their high expression levels in the Atlantic salmon brain suggest functional conservation. Many of the genes downregulated or inactivated in sterile testes have diverse roles, including differentiation of brain regions, maintenance of neural stem cells, neuronal differentiation, neurotransmission and synaptic plasticity, and sensory perception, as well as paracrine and endocrine regulation. Collectively, these findings indicate the presence of shared developmental programs in the brain and normal testes of Atlantic salmon. The downregulation or complete inactivation of the same genes in GC-ablated testes and ovaries underscores their potential importance in reproduction. It is important to note, however, that the microarray platform used in this study does not distinguish between duplicated genes. Therefore, the expression of different paralogs in the gonads and brain cannot be ruled out, and this warrants further investigation.

To the best of our knowledge, transcription signature involved in the fate of GC lineage and the development of brain has not been previously described. Therefore, we can only speculate on its nature, highlighting potential directions for future research. We see three aspects that can be considered: PGC migration and homing, environment of GC residence and differentiation, and entering first sexual maturation. The migration and homing of PGC in both mouse and zebrafish are controlled by chemokines [69,70], cell adhesion molecules, and extracellular matrix interactions [71]. Studies in zebrafish highlighted the role of myosin contraction in PGC in response to chemokine SDF-1 [72]. Similar mechanisms of cell migration are crucial in brain development, ensuring proper neuronal regionalization and the formation of functional networks. It is reasonable to anticipate that the same molecular players can be involved in both gonadal and nervous system development. PGCs possess unique properties that distinguish them from somatic cells, notably their ability to differentiate into any cell type. This developmental potential is orchestrated by a set of genes that regulate cell specification, suppress somatic recombination, maintain genomic integrity, and protect against mutations and transposable element integration. The inactivation of even a single key gene, such as *dnd*, disrupts gametogenesis entirely. In addition to intrinsic regulation, the maintenance of PGCs requires a specialized niche that provides essential signals and structural support to preserve their identity and prevent premature differentiation [73]. The concept of a “niche” emerged from studies on interactions between PGCs and somatic cells in invertebrate models like nematodes and *Drosophila* and has since been extended to other animals and humans, particularly through advances in germline transplantation [74,75,76,77]. Despite growing interest, research in this area remains in its early phase, largely due to the complexity and variability of the PGC microenvironment across developmental stages [76] and species [78]. The inability of cells expressing GC markers and gamete-specific genes to differentiate in spermatozoa indicates that the transcription signature revealed in this study can be essential for establishing the GC niche. Its absence in sterile dnd-deficient testes implies that these genes can be expressed in PGCs, which possess the potential to differentiate into multiple lineages, including neural cells. The increasing application of single-cell sequencing in Atlantic salmon research is expected to clarify this issue by determining whether GC-specific genes and those highly expressed in the brain are co-expressed within the same cellular lineages. Immunohistochemistry or in situ hybridization for neural markers is needed to determine whether these cells form a distinct structure or are dispersed throughout the gonad.

An interesting finding was that genes with high expression in the brain are sharply downregulated in testes at the onset of first sexual maturation. Unlike Pacific salmon of the genus *Oncorhynchus*, which reproduce only once, Atlantic salmon undergo multiple spawning events throughout their lives. The regulation of initial versus subsequent maturations may differ. What triggers the initial decision to enter the reproductive cycle remains unclear, particularly because Atlantic salmon males can mature at markedly different body sizes. While the age of sexual maturity is genetically determined, as demonstrated by successful selective breeding and identification of a master gene *vgll3* [79], the physiological mechanisms mediating this genetic control are unknown. The primary signal of reproductive readiness may originate in the testes, and a provisional neural tissue could, in theory, mediate communication between the gonads and the brain. In contrast to the first maturation, subsequent reproductive cycles occur annually and no longer require a decision point, as the testes come under full external control. Increased daylight stimulates the hypothalamic–pituitary axis (HPA), which triggers the production of sex hormones that drive spermatogenesis.

## 5. Conclusions

A transcriptome comparison between fertile and sterile *dnd*-knockdown in Atlantic salmon, combined with gene expression profiles from normal male gonads, revealed genes involved in sexual maturation and the regulation of this process. While most findings aligned with existing knowledge, several unexpected results were also obtained. The most dramatic effect of *dnd* knockdown was observed in genes with the highest expression in the brain, which are known to play key roles in central nervous system development and function. The origin and role of this transcription signature, which ceases activity at maturation, remains to be investigated.

## Figures and Tables

**Table 1:** PCR primers for RT-qPCR and RT-PCR of selected genes.

Genbank	Gene	Forward Primer	Reverse Primer
RT qPCR			
XM_014143380	*Vasa*	CCCAGTACAGAAGCATGGCA	CACTGGACGCACACAAGTTC
XM_014157064	*Tdrd1*	AGCTCCCTTTCCAGATTGCC	AGCTGTGGCGATGATGGG
XM_014158129	*Tdrkh*	AGGCCAAGGAGCTCATCCTA	CCCCTTCCTCCATCCAGAGA
XM_014171837	*Piwil1*	ATGACATTGCGTGGGACCAT	AGCACGCATCTTGTCAGTCA
XM_014199425	*Dnd1*	TTTGCCTACGCCAAGTACGA	GGAGGCATAGACCACCACTG
XM_014189524	*Tdrd9*	GAACAGGACCATCTGCCACA	CCTCTGAGAAAGGTGCCAGG
RT-PCR			
NM_001165390	*Viaat*	TCGACGTCGCCATATTCGTT	TGGTATGTCGACAGAACCTGC
XM_014139471	*Hr6*	TGGAGAAGAGAAGACGTGCG	AATCCCGCTCTGTACTTGCC
XM_014206751	*Soho*	CATCTCCCAGTTCACCCACC	CCTCTTATGAGTGTGGCCGT
XM_014140645	*Gbh*	TGTCCGATCAACTTGACAACAT	TCTCACAAACAGCTGGCGAA
XM_014148127	*Dbx1*	AGCAAGCACTCCGACTTCTC	CACAACCAATATGGCCCCTAC
NM_001141590	*Crf1*	ACCTGACGTTCCATCTGCTG	GAAAGAACGAAGAAAGTTAACCA
CA049789	*Rpol2*	TAACGCCTGCCTCTTCAGTTGA	ATGAGGGACCTTGTAGCCAGCAA
DW542195	*Eif-3*	CAGGATGTTGTTGCTGGATGGG	ACCCAACTGGGCAGGTCAAGA

**Table 2:** Enrichment of functional groups in genes that were downregulated in sterile testes while maintaining stable expression during normal gonad development.

Functional Group	DEG	Annotation Type
DNA replication	30	GO
Cell cycle	16	KEGG
Gene silencing by RNA	10	GO
DNA repair	36	GO
Double-strand break repair	11	GO
Base-excision repair	6	GO
G1/S transition	22	GO
RNA polymerase	10	KEGG
RNA degradation	14	KEGG
Chromosome	23	GO
Histone binding	17	GO
Pyrimidine metabolism	17	KEGG
Cyclin binding	8	GO
Basal transcription factors	7	KEGG
Mitochondrion	97	GO

DEG—differentially expressed genes.

**Table 3:** Enrichment of functional groups in genes that show increased (upper section) or decreased (lower section) expression during testicular maturation.

Functional Group	DEG	Annotation Type
Upregulated (790 genes) DNA replication	45	GO
Mitosis	21	GO
Meiosis	39	STARS
Cell division	94	GO
Chromosome	41	GO
Chromosome segregation	21	GO
DNA repair	49	GO
Nucleotide excision repair	12	KEGG
Mismatch repair	13	GO
Cilia	48	STARS
Downregulated (689 genes)		
DNA replication	9	KEGG
Homologous recombination	7	KEGG
Mismatch repair	7	KEGG
Peptidase inhibitor activity	25	GO
Carbohydrate binding	22	GO
Metabolism calcium	19	STARS
Growth factor	20	STARS
Midbrain development	10	GO
Notochord development	12	GO
Differentiation homeobox	23	STARS

DEG—differentially expressed genes.

## Data Availability

Microarray data are available in NCBI Geo Omnibus. Available online: https://www.ncbi.nlm.nih.gov/geo/query/acc.cgi?acc=GSE297806 (accessed on 18 August 2025).

## Supplementary Materials

The following supporting information can be downloaded at: https://www.mdpi.com/article/10.3390/genes16091095/s1, Table S1, Genes silenced in gonads of dnd knockdown salmon and during sexual maturation of testes; Table S2, Functional categories of gene ontology enriched in genes that were silenced in gonads of dnd knockdown in salmon.

## Abbreviations

The following abbreviations are used in this manuscript:

DEG	differentially expressed genes
ER	expression ratio
GC	germ cells
HPA	hypothalamic–pituitary–adrenal (HPA) axis
PGC	primordial germ cells
